# CBP/p300 HAT maintains the gene network critical for β cell identity and functional maturity

**DOI:** 10.1038/s41419-021-03761-1

**Published:** 2021-05-12

**Authors:** Linlin Zhang, Chunxiang Sheng, Feiye Zhou, Kecheng Zhu, Shushu Wang, Qianqian Liu, Miaomiao Yuan, Zhaoqian Xu, Yun Liu, Jieli Lu, Jianmin Liu, Libin Zhou, Xiao Wang

**Affiliations:** 1 Department of Endocrine and Metabolic Diseases, Shanghai Institute of Endocrine and Metabolic Diseases, Ruijin Hospital, Shanghai Jiao Tong University School of Medicine, Shanghai, China; 2 Shanghai National Clinical Research Center for Metabolic Diseases, Key Laboratory for Endocrine and Metabolic Diseases of the National Health Commission of the PR China, Shanghai National Center for Translational Medicine, Ruijin Hospital, Shanghai Jiao Tong University School of Medicine, Shanghai, China

**Keywords:** Acetylation, Type 2 diabetes

## Abstract

Loss of β cell identity and functional immaturity are thought to be involved in β cell failure in type 2 diabetes. CREB-binding protein (CBP) and its paralogue p300 act as multifunctional transcriptional co-activators and histone acetyltransferases (HAT) with extensive biological functions. However, whether the regulatory role of CBP/p300 in islet β cell function depends on the HAT activity remains uncertain. In this current study, A-485, a selective inhibitor of CBP/p300 HAT activity, greatly impaired glucose-stimulated insulin secretion from rat islets in vitro and in vivo. RNA-sequencing analysis showed a comprehensive downregulation of β cell and α cell identity genes in A-485-treated islets, without upregulation of dedifferentiation markers and derepression of disallowed genes. A-485 treatment decreased the expressions of genes involved in glucose sensing, not in glycolysis, tricarboxylic acid cycle, and oxidative phosphorylation. In the islets of prediabetic *db/db* mice, CBP/p300 displayed a significant decrease with key genes for β cell function. The deacetylation of histone H3K27 as well as the transcription factors Hnf1α and Foxo1 was involved in CBP/p300 HAT inactivation-repressed expressions of β cell identity and functional genes. These findings highlight the dominant role of CBP/p300 HAT in the maintenance of β cell identity by governing transcription network.

## Introduction

It is well established that type 2 diabetes is generally due to the progressive decline of islet β cell function, with loss of β cell identity^1^. β cell identity is usually characterized by the continuous expression of β cell-lineage genes and the sustained repression of non-β cell program^2^. In mice, the ablations of transcription factors such as Pdx1, MafA, Nkx6.1, Pax6, NeuroD1, and Foxo1, lead to loss of β cell identity by repressing β cell functional genes and inducing genes disallowed in β cells^1,3–7^. It has been demonstrated that hyperglycemia, oxidative stress, and inflammation inhibit transcription factor activities and perturb β cell identity in mice and human^8–10^. However, the mechanism underlying the intrinsic transcription networks-mediated the maintenance of β cell identity remains largely understood.

Chromatin modification plays an essential role in specific gene expression programs of differentiated cells^11^. Histone acetylation is widely regarded as an active epigenetic marker of gene transcription through unwrapping the interaction between DNA and chromatin and enhancing the chromatin accessibility^12^. Of interest, acetylation of histone H3 lysine 27 (H3K27Ac) is a hallmark of active enhancers^13^, which are highly associated with cell type-specific gene expression patterns^14^. Histone acetylation is determined by competing activities of histone acetyltransferases (HATs) and histone deacetylases (HDACs). Among the HATs members, CREB-binding protein (CBP) and p300 are essential for H3K27Ac^15^. CBP and p300 act as multifunctional transcriptional co-activators with extensive sequence homology and functional similarity. Both CBP and p300 possess a HAT domain and several protein-protein interaction domains^16^, which make them prone to serve as transcriptional scaffolds for recruiting a number of transcription factors^17^.

It has been reported that CBP and p300 synergistically promote insulin gene transcription by interacting with transcription factors NeuroD1, Pdx1, and Klf11^18–21^. p300 is recruited to Glut2 gene promoter and coactivates Glut2 expression via the interaction with transcription factor Hnf1α^22^. p300 also facilitates Klf11-regulated Pdx1 transcription in β cells^23^. Mice with an activating mutant CBP (S436A) display increased β cell proliferation but diminished glucose-stimulated insulin secretion (GSIS)^24^. Triallelic knockout of CBP/p300 (CBP^Het^; p300^KO^) in pancreatic endocrine progenitors impairs proliferation of neonatal α and β cells in mice^25^. These data implicate an essential regulatory role of CBP and p300 in islet β cells. However, it is still unclear whether the HAT domain is crucial for CBP/p300-regulated β cell function.

A-485 is a newly discovered selective inhibitor of CBP/p300 HAT activity through bounding to the catalytic active site of CBP/p300 and competing with acetyl coenzyme A, displaying 1000-fold more potent than previously described control compounds including C646^26^. In this current study, A-485 treatment markedly suppressed GSIS in rat islets in vitro and in vivo. RNA sequencing (RNA-seq) revealed a comprehensive down-regulation of β cell identify genes in CBP/p300 HAT-inhibited rat islets. ChIP sequencing **(**ChIP-seq) analysis provided insight into the involvement of H3K27 acetylation in CBP/p300 HAT-governed transcription networks of islets.

## Results

### Inhibition of CBP/p300 HAT diminishes insulin secretion and reprograms transcription networks in rat islets

**Fig. 1:**
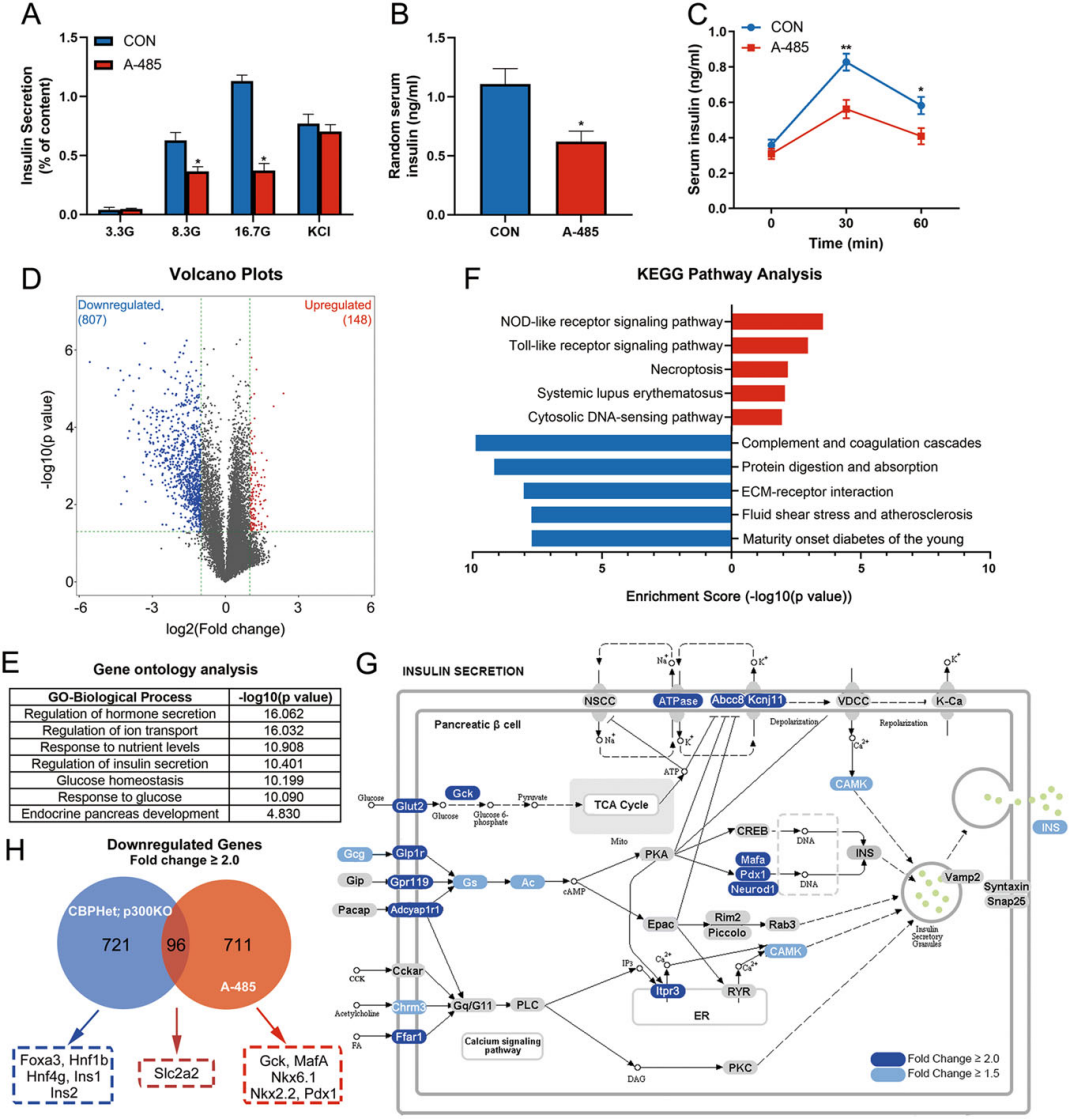
Effects of A-485 on insulin secretion and transcriptome profile of rat islets. **A** Isolated rat islets were pretreated with 3 μM A-485 at 5.6 mM glucose for 16 h, followed by stimulation with 3.3, 8.3 and 16.7 mM glucose or 35 mM KCl for 1 h, and insulin secretion was measured. **B** Random serum insulin levels of control and A-485-treated C57BL/6 mice (*n* = 5). **C** Serum insulin levels under glucose loading in control and A-485-treated C57BL/6 mice (*n* = 5). **D** Volcano plots of differentially expressed genes. **E** Top 5 upregulated (red) and downregulated (blue) pathways in KEGG analysis. **F** GO analysis of downregulated genes. **G** Visualization of downregulated genes involved in insulin secretion pathway. Dark blue represents fold change ≥2.0 and light blue represents fold change ≥1.5. Gray-labeled genes show no significant change. **H** Overlap analysis of downregulated genes between CBP/p300 HAT-inhibited rat islets and pancreatic endocrine progenitors-specific CBP^Het^; p300^KO^ mice islets (fold change ≥2.0, *p*-value <0.05). Data are given as mean ± SD for three separate experiments. *p < 0.05, **p < 0.01 *vs* control (CON) group.

To investigate the effect of CBP/p300 HAT on β cell function, isolated rat islets were pretreated with A-485 for 16 h, and then stimulated with various concentrations of glucose or 35 mM KCl for 1 h. As shown in Fig. 1A, A-485 pretreatment significantly suppressed insulin secretion from islets in response to glucose, but without effect on the stimulation of secretion provoked by KCl. Consistent with the in vitro result, random serum insulin level and GSIS were significantly decreased in A-485-treated mice compared with control mice (Fig. 1B, C). These data indicate that HAT domain of CBP/p300 is important for its regulation on islet β cell function.

To elucidate the molecular mechanism underlying CBP/p300 HAT-governed islet function, RNA-seq analysis was performed in A-485-treated rat islets. A total of 955 differentially expressed genes were identified between control and A-485 groups (fold change ≥2.0, *p* < 0.05), among which 807 genes were downregulated while only 148 genes were upregulated (Fig. 1D). Gene Ontology (GO) analysis revealed that A-485-downregulated genes were associated with β cell essential biological processes, such as regulation of insulin secretion, response to glucose, and endocrine pancreas development (Fig. 1E). KEGG pathway analysis further showed that maturity onset diabetes of the young (MODY) pathway including a series of β cell essential transcription factors and functional genes, was one of the most profound pathways downregulated by CBP/p300 HAT inhibition (Fig. 1F). The genes important for fuels metabolism, secretory pathway, insulin gene transcription, and ion transport in insulin secretion pathway were extensively downregulated by A-485 (Fig. 1G).

We further analyzed the overlap of downregulated genes between A-485-treated islets and pancreatic endocrine progenitors-specific CBP/p300 triallelic knockout (CBP^Het^; p300^KO^) islets^25^. Amazingly, only 11.9% of A-485-repressed genes (96/807) were uniformly downregulated in CBP^Het^; p300^KO^ islets (Fig. 1H). Gck, Pdx1, MafA, Nkx6.1, and Nkx2.2 were only repressed in A-485-treated islets, not in the triallelic knockout ones. These results manifest the dominant role of HAT in CBP/p300-governed β cell transcription networks.

### β cell and α cell identity genes are repressed by CBP/p300 HAT inhibition

**Fig. 2:**
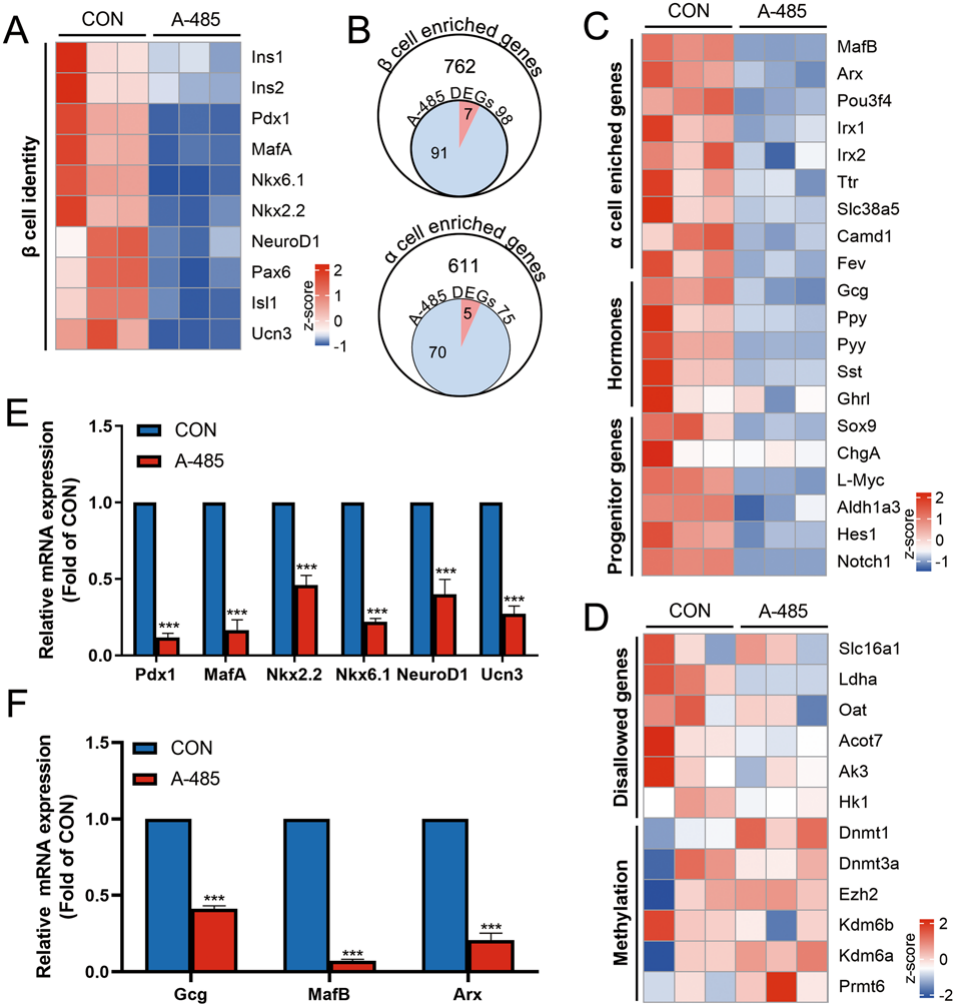
CBP/p300 HAT inhibition leads to reduced expressions of β and α identity genes. **A** Heatmap of β cell identity genes. **B** Venn diagrams of upregulated (red) and downregulated (blue) genes enriched in β and α cell by A-485 treatment. **C** Heatmap of α cell identity genes, islet hormone genes, and progenitor markers. **D** Heatmap of disallowed genes and related methylases and demethylases. **E**–**F** Isolated rat islets were treated with 3 μM A-485 for 16 h and mRNA levels of β and α cell identity genes were detected by RT-qPCR. Data are given as mean ± SD for three separate experiments. ***p < 0.001 vs control group.

As aforementioned, loss of β cell identity is tightly linked to β cell dysfunction. Surprisingly, our RNA-seq data revealed a comprehensive repression of β cell identity genes in A-485-treated rat islets (Fig. 2A). β cell identity-determining transcription factors (i.e. Pdx1, MafA, Nkx6.1, Nkx2.2, NeuroD1, Pax6 and Isl1^27^), as well as Ucn3, a mature β cell marker^28^, were all conspicuously downregulated following CBP/p300 HAT inhibition. In the gene set identified by Qiu et al.^29^, we found that 98 β cell enriched genes were regulated by A-485, among which 92.9% were significantly downregulated (Fig. 2B and Table S1), indicating loss of β cell identity.

It has been reported that loss of β cell identity is usually accompanied by β cell transdifferentiation towards other islet cell types or dedifferentiation towards a fetal-like state^1,5,6^. However, 93.3% of 75 genes highly expressed in α cell lineage^29^ were significantly downregulated in rat islets by A-485 treatment (Fig. 2B, C and Supplementary Table S2). Multiple islet hormone genes such as Ppy, Pyy, Sst, and Ghrl, were also repressed by A-485 (Fig. 2C). This was the case for the dedifferentiation markers including Sox9, ChgA, L-Myc, and Aldh1a3^30^ (Fig. 2C).

Ectopic expression of disallowed genes is regarded as an important feature of loss of β cell identity, which is closely related to DNA and histone methylation^31^. We checked the expressions of two sets of disallowed genes identified by Pullen et al.^32^ and Thorrez et al.^33^, and found no aberrant derepression for these genes in A-485-treated rat islets (Fig. 2D and Supplementary Fig. S1). Nor did their regulators methylases and demethylases^34–36^ (Fig. 2D). These data figure that inhibition of CBP/p300 HAT results in loss of β cell identity without returning to an immature state or reprogramming to other islet cell types. Conversely, CBP/p300 HAT activity may be essential for the continuous expression of identity genes in multiple islet cell types, not just in islet β cell.

**Fig. 3:**
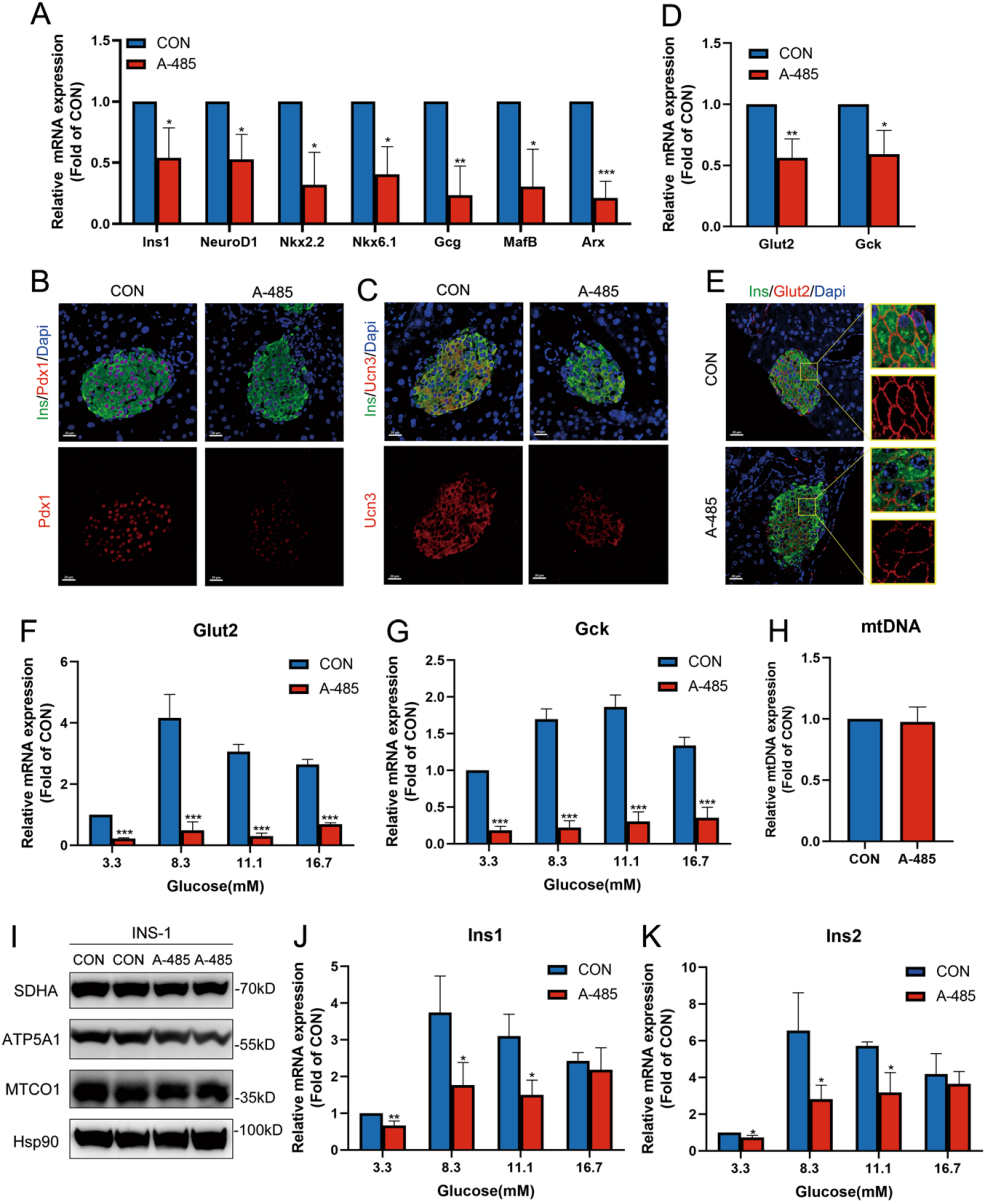
Impaired β cell identity and functional maturity in CBP/p300 HAT-inhibited β cell. **A** mRNA expressions of β and α cell identity genes in isolated islets from control and A-485-treated mice. **B**–**C** Representative pancreatic sections co-immunostained for insulin (Ins, green), Pdx1 or Ucn3 (red) and DAPI (blue) from control and A-485-treated mice (scale bars, 20 μm). **D** mRNA expressions of Glut2 and Gck in isolated islets from control and A-485-treated mice. **E** Representative pancreatic sections co-immunostained for insulin (Ins, green), Glut2 (red), and DAPI (blue) from control and A-485-treated mice (scale bars, 20 μm). **F**–**G** mRNA expressions of Glut2 and Gck at various concentrations of glucose in isolated rat islets treated with A-485 for 16 h. **H** mtDNA expression in control and A-485-treated INS-1 cells for 16 h. **I** Protein expressions of mitochondrial MTCO1, ATP5A1 and SDHA in control and A-485-treated INS-1 cells for 16 h. **J**–**K** mRNA levels of Ins1 and Ins2 at various concentrations of glucose in isolated rat islets treated with A-485 for 16 h. Data are given as mean ± SD for three separate experiments. *p < 0.05, **p < 0.01, ***p < 0.001 *vs* control group.

The expressions of islet identity genes were further validated by real-time quantitative PCR (RT-qPCR) under the condition of CBP/p300 HAT inhibition. Consistent with the result of RNA-seq, mRNA expressions of β and α cell identity genes (Pdx1, MafA, Nkx2.2, Nkx6.1, NeuroD1, Ucn3, Gcg, MafB, Arx) were lowered in A-485-treated rat islets (Fig. 2E, F) and isolated islets from mice injected with A-485 (Fig. 3A). Pdx1 and Ucn3 protein expressions exhibited a similar decrease as shown by immunostaining (Fig. 3B, C).

### CBP/p300 HAT inhibition impairs the glucose sensing of islet β cells

A mature pancreatic β cell is defined as an insulin-secreting cell sensitive to glucose stimulation^37^. CBP/p300 HAT activity is essential for β cell function maturity as A-485 treatment leads to diminished GSIS (Fig. 1A–C). We explored the molecular pathways dominated by CBP/p300 HAT. Glut2 and Gck mRNA expressions were significantly decreased in islets of A-485-treated mice compared with those of control mice (Fig. 3D). Immunostaining showed a similar result for Glut2 protein expression (Fig. 3E). In line with the in vivo results, A-485 treatment also inhibited Glut2 and Gck mRNA expressions at various concentrations of glucose in isolated rat islets (Fig. 3F, G). However, the expressions of other genes involved in glycolysis, pentose phosphate pathway, TCA cycle, and oxidative phosphorylation revealed no obvious changes after A-485 treatment (Fig. S2). Nor did the expressions of mitochondrial mtDNA and key enzymes in INS-1 cells treated with A-485 (Fig. 3H, I). These data indicate that the GSIS impaired by CBP/p300 HAT inhibition is attributed to less glucose influx mediated by Glut2 and Gck in islet β cells.

In addition, A-485 decreased Ins1 and Ins2 expressions at 3.3, 8.3, and 11.1 mM glucose in isolated rat islets (Fig. 3J, K). However, the inhibitory action of A-485 on insulin gene transcription disappeared when the glucose concentration was elevated to 16.7 mM (Fig. 3J, K).

### CBP/p300 HAT regulates the expression of Hadh responsible for basal insulin secretion

**Fig. 4:**
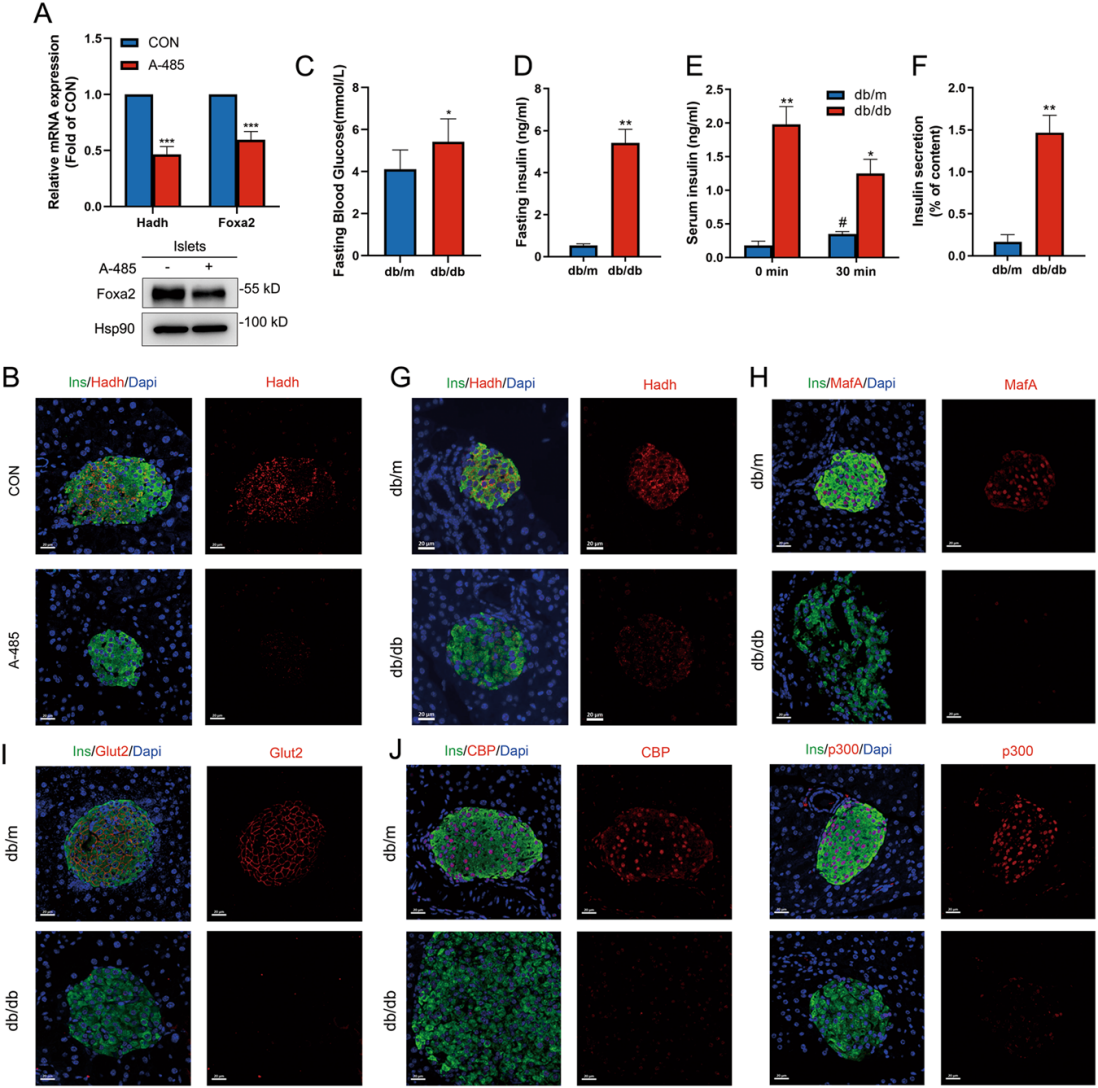
CBP/p300 HAT mediates expression of Hadh responsible for basal insulin secretion. **A** mRNA and protein levels of Hadh and Foxa2 in isolated rat islets treated with A-485 for 16 h. **B** Representative pancreatic sections co-immunostained for insulin (Ins, green), Hadh (red), and DAPI (blue) from control and A-485-treated C57BL/6 mice (scale bars, 20 μm). **C** Blood glucose levels of 4-week *db/m* and *db/db* mice were measured after 16 h fasting. **D** Fasting serum insulin levels of 4-week *db/m* and *db/db* mice. **E** Serum insulin levels after glucose loading in 4-week *db/m* and *db/db* mice (*n* = 5). **F** Isolated islets from 4-week-old *db/m* and *db/db* mice were stimulated with 5.6 mM glucose for 1 h to measure insulin secretion. **G**–**J** Representative pancreatic sections co-immunostained for insulin (Ins, green), Hadh/MafA/Glut2/CBP/p300 (red), and DAPI (blue) from 4-week-old *db/m* and *db/db* mice (scale bars, 20 μm). Data are given as mean ± SD. * *p* < 0.05, **p < 0.01, ***p < 0.001 *vs* control group, #*p* < 0.001 *vs* 0 min.

Neonatal β cells display immature functional characteristic, with a high basal insulin secretion at low glucose concentrations and small fold increase in insulin secretion with high glucose stimulation^28^. A cluster of genes, whose mutations lead to congenital hyperinsulinism (CHI) in human, are responsible for the regulation of insulin secretion under basal condition^38^. Attractively, our RNA-seq showed that three inactivating-mutated pathogenic genes of CHI, including ATP-sensitive potassium channel subunits Kcnj11, Abcc8, and hydroxyacyl-coenzyme A dehydrogenase (Hadh), were obviously repressed by A-485 (Fig. S3). Hadh catalyzes the third step of fatty acid β-oxidation cycle for medium/short-chain 3-hydroxyacyl-CoAs and is highly expressed in islet β cells^39^. As shown in Fig. 4A, mRNA level of Hadh was markedly decreased in A-485-treated rat islets. Foxa2, an essential transcription factor for Hadh expression^40^ also displayed lowered mRNA and protein levels after A-485 treatment. Similarly, Hadh protein level was also declined in islets of mice treated with A-485 (Fig. 4B).

In the incipient stage of type 2 diabetes, there is a transient compensatory rise in insulin secretion from pancreatic β cells to meet the increased metabolic demands as in patients with impaired glucose tolerance and *ob/ob* mice^41^. In 4-week-old, *db/db* mice displayed a slight increase in fasting blood glucose level as compared with the lean controls (Fig. 4C), with steeply elevated fasting serum insulin but severely impaired GSIS (Fig. 4D, E). Islets isolated from 4-week *db*/*db* mice also exhibited basal insulin hypersecretion (Fig. 4F), similar to those from Hadh-KO mice^42^ and immature islet β cells^43^. Hadh protein expression was significantly attenuated in islets of 4-week-old *db*/*db* mice compared with *db/m* mice (Fig. 4G), along with decreased expressions of MafA and Glut2 (Fig. 4H, I). In parallel with these results, CBP and p300 abundances were also dramatically declined in 4-week-old *db*/*db* mice (Fig. 4J). These results indicate that loss of β cell identity and impaired functional maturity already occur in 4-week *db/db* mice, in which decreased CBP/p300 expression might be involved.

### Role of H3K27Ac in CBP/p300 HAT-governed transcription networks of islets

**Fig. 5:**
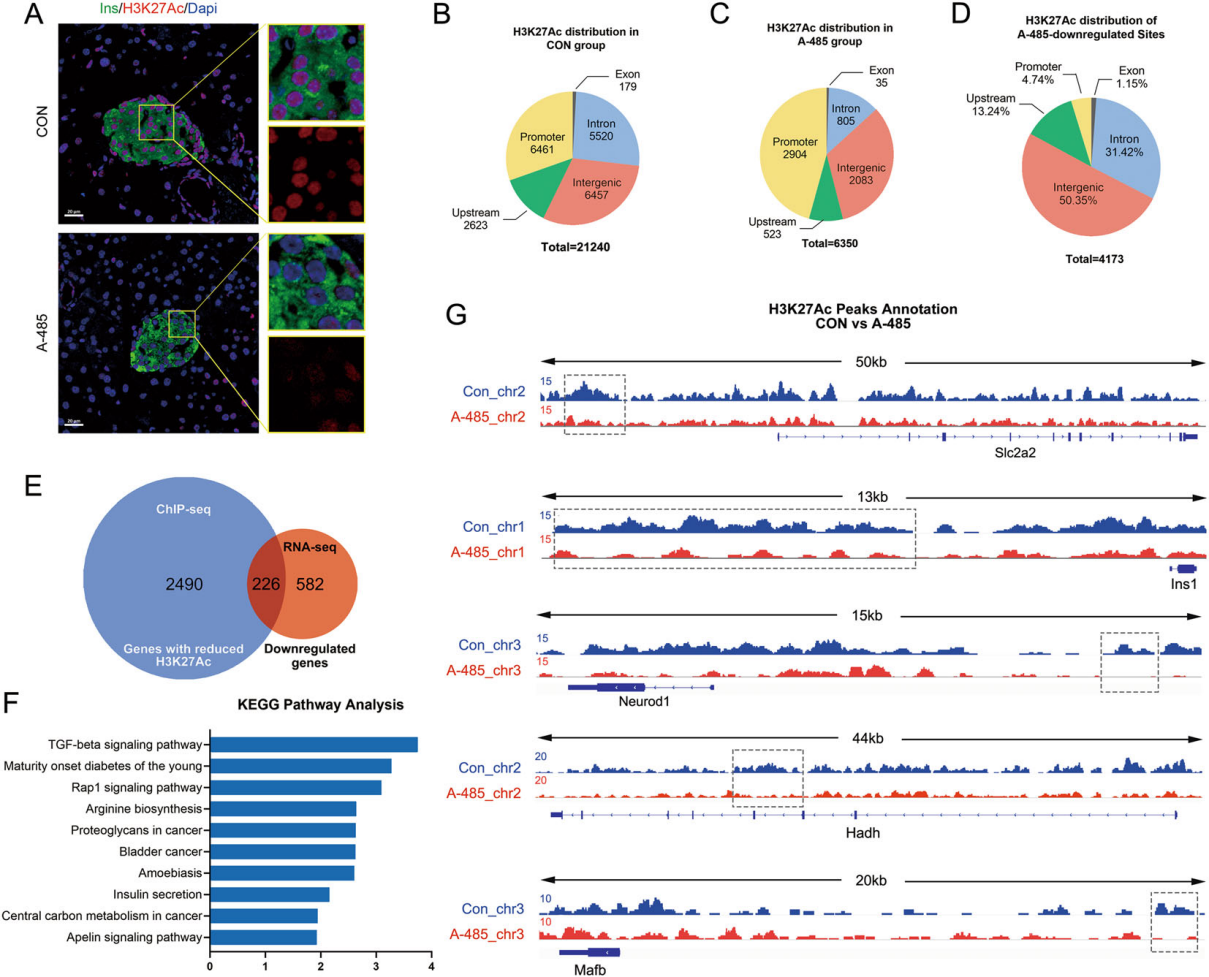
Role of H3K27Ac in CBP/p300-mediated islet identity gene expressions. **A** Representative pancreatic sections co-immunostained for insulin (Ins, green), H3K27Ac (red), and DAPI (blue) from control and A-485-treated mice (scale bars, 20 μm). **B**–**C** Genomic distribution of H3K27Ac peaks in control and A-485-treated rat islets. **D** Genomic distribution of downregulated H3K27Ac peaks between two groups (fold change ≥2.0, *p* value <0.001). **E** Overlap analysis of annotated genes with hypo-H3K27Ac and downregulated genes identified in RNA-seq. **F** KEGG pathway analysis of overlapping genes. **G** Visualization of H3K27ac levels in adjacent area of transcriptional regions. Chromosomal span and gene structure are shown, and the significantly downregulated peaks are highlighted with the gray dotted box.

H3K27Ac is reported to be a biomarker of cell type-specific promoters and enhancers related to key cell identity gene expressions, which is mainly maintained by the acetyltransferase CBP/p300^15^. As expected, H3K27Ac abundance was significantly reduced in islets of A-485-treated mice compared with that of control mice (Fig. 5A). To determine the role of H3K27Ac in CBP/p300 HAT-dominated islet transcription networks, we performed ChIP-seq analysis in A-485-treated rat islets using anti-H3K27Ac antibody. A total of 21 240 H3K27Ac peaks were identified in control islets (Fig. 5B) while only 6 350 in A-485-treated islets (Fig. 5C), presenting a sharp decline of H3K27Ac level due to CBP/p300 HAT inhibition. By setting a threshold at fold change ≥2.0, *p-*value <0.001, 4 173 downregulated sites were further identified between two groups, more than half of which were distributed in the intergenic region (Fig. 5D).

The downregulated H3K27Ac peaks were annotated by 2 716 genes, which were submitted to overlap analysis with 807 downregulated genes identified by RNA-seq. Surprisingly, only 226 downregulated genes exhibited hypoacetylation of H3K27 under the condition of CBP/p300 HAT inhibition (Fig. 5E). For the overlapping genes, KEGG pathway analysis showed significant enrichment in MODY pathway and insulin secretion pathway (Fig. 5F). Several β cell identity and functional genes (i.e., NeuroD1, Ins1, Glut2, and Hadh) as well as α cell specific transcription factor MafB displayed marked hypoacetylation in their upstream or intron regions after A-485 treatment (Fig. 5G), implicating a link of H3K27Ac to CBP/p300 HAT-mediated β and α cell-lineage gene expressions. However, the majority of downregulated genes, including β cell identity genes (i.e., Pdx1, Nkx6.1 and MafA), had no alternation in H3K27Ac level after CBP/p300 HAT inhibition, indicating the involvement of alternative mechanisms in CBP/p300 HAT-regulated β cell intrinsic transcription networks.

### Transcription factors are associated with CBP/p300 HAT-mediated gene expression

**Fig. 6:**
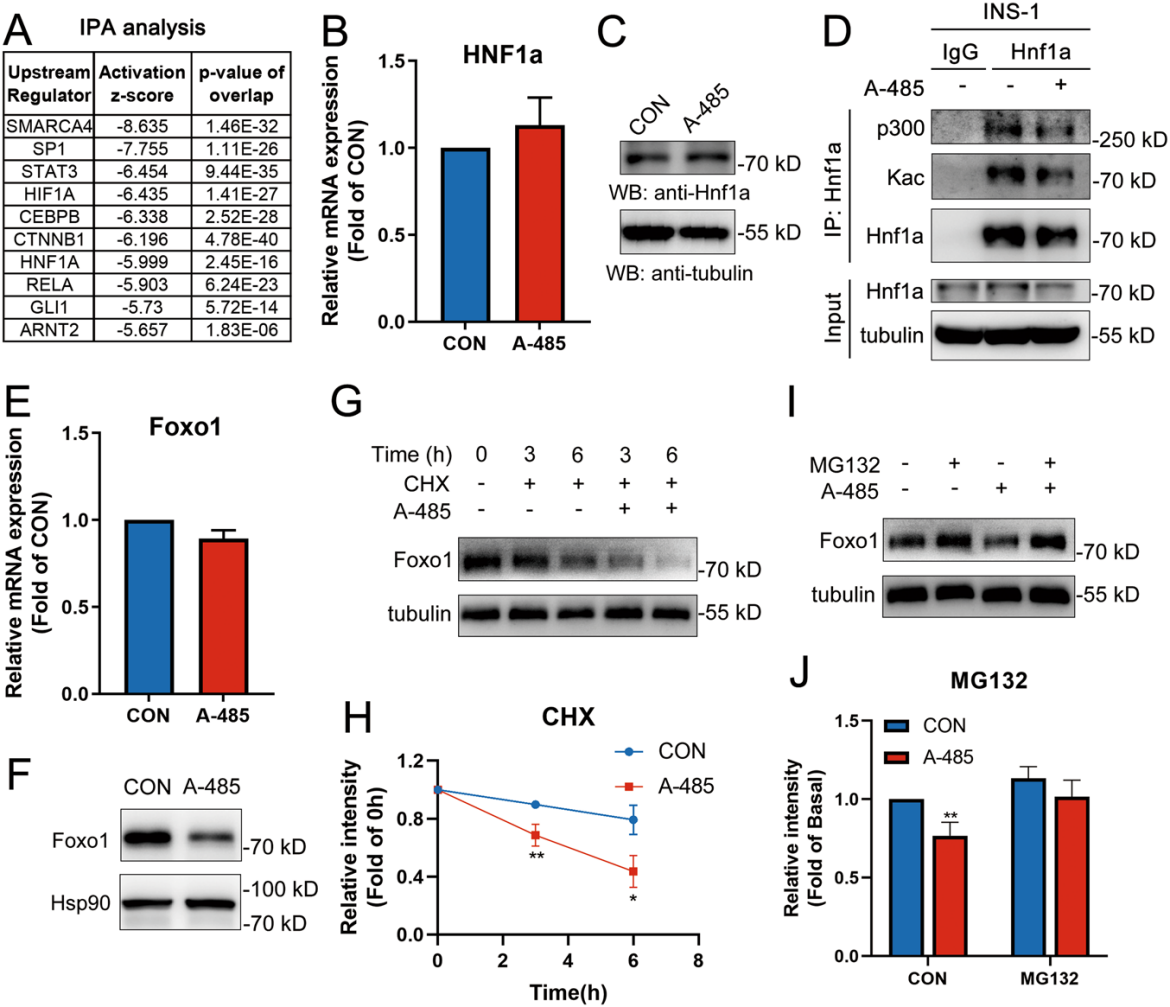
Transcription factors Hnf1α and Foxo1 are regulated by CBP/p300 HAT activity. **A** Top 10 of upstream transcriptional regulators predicted by IPA analysis based on the downregulated gene set identified in RNA-seq. **B**–**C** mRNA and protein levels of Hnf1α in isolated rat islets treated with A-485 for 16 h. **D** Co-immunoprecipitation using Hnf1α antibody in A-485 treated INS-1 cells for 6 h. **E**–**F** mRNA and protein levels of Foxo1 in A-485-treated rat islets for 16 h. **G**–**H** Foxo1 levels in INS-1 cells treated with 3 μM A-485 and 10 μg/ml cycloheximide (CHX) for the indicated time. Signal intensity was quantified by Image J software for statistical comparison. **I**–**J** Foxo1 levels in INS-1 cells treated with 3 μM A-485 and 10 μM MG132 for 6 h. Signal intensity was quantified by Image J software for statistical comparison. Data are given as mean ± SD. *p < 0.05, **p < 0.01 *vs* control group.

In order to investigate the non-histone mechanism of CBP/p300 HAT, the prediction of upstream transcription factor was performed using Ingenuity Pathway Analysis (IPA) based on the downregulated gene set identified by RNA-seq. Attractively, Hnf1α, a possible target of CBP and p300 in mice islets^25^, ranked in top 10 inhibited transcription regulators in IPA analysis (Fig. 6A). It has been demonstrated that Hnf1α recruits CBP and p300 for effective transactivation^22,44^, and loss of Hnf1α in mice results in decreased expressions of genes crucial for islet β cell function, including Ins1, Pdx1, NeuroD1, and Glut2^45^. However, there was no significant alteration in mRNA and protein levels of Hnf1α in A-485-treated islets (Fig. 6B, C). In consideration of the intrinsic HAT activity of CBP/p300, we detected Hnf1α acetylation level in INS-1 cells. A-485 treatment did decrease acetylation of endogenous Hnf1α. Moreover, the interaction of Hnf1α and p300 was markedly attenuated by A-485 (Fig. 6D). Thus, it is likely that CBP/p300 HAT-mediated acetylation of Hnf1α is associated with its co-activation for target gene expressions in islet β cell.

In addition, Foxo1 is widely accepted as an identity determinant gene for islet β cells^1^, protecting β cells when facing the metabolic stress partially through inducing NeuroD1 and MafA expressions^46^. Foxo1 mRNA expression was without significant change in A-485-treated rat islets (Fig. 6E), but its protein abundance displayed a marked decline (Fig. 6F). Given that Foxo1 could be acetylated by CBP/p300 and its deacetylation leads to decreased stability via ubiquitination and followed proteasomal degradation^47^, we detected the protein level of Foxo1 in INS-1 cells treated with A-485 and the protein synthesis inhibitor CHX. A-485 treatment did result in shortened half-life of Foxo1 in INS-1 cells in the presence of CHX (Fig. 6G, H). A-485-promoted protein degradation of Foxo1 was abolished by the proteasome inhibitor MG132 (Fig. 6I, J). These findings suggest that Foxo1 may also be involved in CBP/p300 HAT-mediated gene expression in β cells.

## Discussion

In islet β cell, CBP/p300 is usually known to be a co-activator of transcriptional factors such as Pdx1, NeuroD1, Klf11, and Hnf1α to regulate key β cell functional gene expressions^19,22,23^. In this current study, the inhibition of CBP/p300 HAT by A-485 resulted in a decrease in GSIS, with comprehensive repression of β cell identity and functional gene expressions. Inactivation of CBP/p300 HAT by A-485 displayed more comprehensive suppression on β cell functional genes than CBP/p300 triallelic knockout^25^, indicating a compensatory effect of the residual single allelic and the redundancy of the four copies of CBP/p300 in the regulation of β cell transcription networks. Therefore, mutation or deletion of all four copies of CBP/p300 HAT domain in adult β cells will provide more in-depth insight into the mechanism underlying CBP/p300 HAT-maintained β cell-specific gene expression pattern.

Loss of β cell identity is tightly linked to β cell failure in type 2 diabetes mellitus^48^. It has been demonstrated that continual activation of several β cell-enriched transcription factors, including Foxo1, Pdx1, MafA, NeuroD1, Nkx2.2, Nkx6.1, and Pax6, is crucial for the maintenance of β cell identity^1,3–7,49^. Surprisingly, these identity-determining transcription factors were all markedly repressed by A-485, implying that CBP and p300 may function as superior regulators for the maintenance of β cell identity mediated by its HAT activity. Several clinical studies have found evidence of β cell dedifferentiation in pancreatic islets of patients with type 2 diabetes mellitus^48,50,51^. Chronic glucotoxicity^8^, endoplasmic reticulum stress^52^, oxidative stress, hypoxia^9^, and inflammation^10^ have been demonstrated to be involved in β cell dedifferentiation. Loss of β cell identity is usually accompanied by the dedifferentiation towards a progenitors-like state^1,4,6^ and the transdifferentiation to other islet cell types^3,5,7,34,49^. However, like β cell identity genes, markers of progenitor cells and other kinds of hormone-secreted cells were all downregulated in the condition of CBP/p300 HAT inhibition. p300 and CBP enhance transactivation on the glucagon gene by interacting with transcription factor Pax6^53^. The proliferation of neonatal α cells was reduced in p300-null islets^25^. Consistent with these results, inhibition of CBP/p300 HAT by A-485 suppressed the expressions of α cell lineage genes, including Gcg, MafB, and Arx. Continuous inhibition of disallowed genes is also crucial for the maintenance of β cell identity^54^. In this study, no aberrant derepression of disallowed gene expressions was observed in CBP/p300 HAT-inhibited rat islets. These results suggest that the HAT activity of CBP/p300 might act as an identity guardian for multiple cell types of pancreatic islet, not just for islet β cells.

It has been reported that CBP/p300 interact with transcription factors such as Pdx1, NeuroD1, and Klf11 to coactivate insulin gene transcription^18,19,21^. Ins1 and Ins2 mRNA expressions were significantly decreased in CBP/p300 triallelic knockout islets, not in biallelic knockout islets^25^. Our study also displayed an obvious downregulation in this gene in A-485-treated islets, implicating a dominant role of HAT in CBP/p300-mediated Ins gene expression. Our study also figured out the importance of CBP/p300 HAT activity in the expressions of other islet hormones. Moreover, CBP/p300 HAT had a strong action on the expressions of MODY genes. p300 has a coregulatory activity among 90% of MODY genes, including MODY1 (Hnf4α), MODY2 (Gck), MODY3 (Hnf1α), MODY4 (Pdx1), MODY5 (Hnf1β), MODY6 (NeuroD1/Beta2), and MODY7 (Klf11), which regulate islet-enriched gene expression in a coordinated manner^23^. Klf11 interacts with the coactivator p300 to mediate Pdx1 activation^24^. Pdx1 interacts with p300 to activate target genes in pancreatic β cells, including insulin gene^55^. NeuroD1/Beta2, Hnf4α, and Hnf1α gene mutations have been reported to impair their interactions with p300^56,57^. In our study, MODY genes Pdx1, NeuroD1, Gck, Abcc8, and Kcnj11 were downregulated by A-485, indicating that CBP/p300 are not only coactivators of MODY genes, but also their transcriptional activators.

A mature pancreatic β cell is sensitive to glucose stimulation^37^. Glut2 and Gck are two glucose sensors in β cells. Glucose phosphorylation by Gck is the rate-limiting for glucose metabolism to trigger insulin secretion^58^. In this study, A-485 treatment decreased Glut2 and Gck expressions, without altering the expressions of genes related to glycolysis and oxidative phosphorylation. These results suggest that the inhibition of CBP/p300 HAT leads to decreased glucose-stimulated insulin secretion mainly via attenuating glucose flux into β cells, rather than directly disrupting glucose oxidation. Inappropriate basal insulin secretion is a feature of functional immature β cells^28,43^. However, little is known about the negative regulation of basal insulin secretion. Inactivating mutation of Hadh gene gives rise to basal and non-glucose-stimulated hyperinsulinemia in both clinical cases^59^ and gene defect mice^42^. In the present study, A-485 treatment inhibited Hadh expression in rat islets in vitro and in vivo. Pre-diabetic 4-week-old *db/db* mice displayed simultaneous repressions of Hadh, Glut2 and MafA as well as CBP/p300. These results implicate a possible role of Hadh in basal insulin hypersecretion of pre-diabetic status. CBP/p300 may be involved the decreased expressions of β cell functional genes in the development of type 2 diabetes.

Mechanistically, H3K27Ac enrichment has been established as an active marker in cell type-specific enhancer elements^13^, which is associated with islet functional gene expressions^60^. However, to what extent the change of H3K27 acetylation affects the expression of β cell identity genes is not well established. Surprisingly, only 226 of 807 genes downregulated by A-485 displayed decreased levels of H3K27Ac, including β and α cell identity genes Ins1, Slc2a2, NeuroD1, and MafB. H3K27Ac levels of many other islet identity genes repressed by CBP/p300 HAT inhibition were unaffected, suggesting an involvement of alternative mechanisms. More than 100 non-histone transcription-regulating proteins, including transcriptional factors, transcriptional co-activators, and nuclear receptors, are regulated by acetylation^55^. Interestingly, our study revealed the involvement of acetylated Hnf1α and Foxo1 in CBP/p300 HAT-mediated gene expression. A-485 disrupted the interaction of Hnf1α and p300 through downregulating the acetylation level of Hnf1α, which might attenuate their co-activation for target gene expressions in islet β cells. In addition, inhibited CBP/p300 HAT activity accelerated Foxo1 protein degradation, indicating another non-histone acetylation mechanism underlying CBP/p300 HAT-mediated transcription network.

**Fig. 7:**
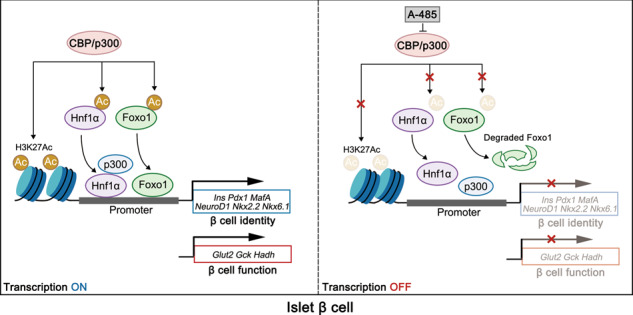
The schematic illustration summarizes CBP/p300 HAT-governed transcription network in islet β cell. The HAT activity of CBP/p300 is required for maintaining the expressions of β cell identity genes and functional genes partially via acetylating histone H3K27 as well as transcription factors Hnf1a and Foxo1. Inhibition of CBP/p300 HAT by A-485 leads to the deacetylation of H3K27, which restricts chromatin accessibility. In addition, inactivation of CBP/p300 HAT also decreases the acetylation levels of Hnf1α and Foxo1, repressing the transcription activation of the target genes by preventing the interaction of Hnf1α and p300 as well as promoting Foxo1 protein degradation.

In summary, our work highlights the dominant role of CBP/p300 HAT activity in the maintenance of β cell identity and functional maturity. The inhibition of CBP/p300 HAT by A-485 leads to impaired islet function and decreased expressions of islet cell identity genes. In the islets of prediabetic *db*/*db* mice, CBP/p300 display a significant decrease with key β cell function genes. CBP/p300 are essential for the expressions of β cell identity and functional genes by acetylating H3K27 as well as the transcription factors Hnf1α and Foxo1, and establishing a HAT-governed hierarchical transcription network (Fig. 7).

## Methods and materials

### Animals

8-week-old male Sprague-Dawley rats and 8-week-old male C57BL/6 mice were obtained from Shanghai Slack Experimental Center. 4-week-old male *db/db* mice and littermate lean *db/m* mice were purchased from Beijing Vital River Laboratory Animal Technology Co., Ltd. C57BL/6 mice were intraperitoneally injected with A-485 (20 mg kg^−1^ day^−1^) for 7 days. All the animals were housed under 12/12 h light/dark cycles with *ad libitum* feeding, in full accordance with the protocols approved by the Animal Care Committee of Ruijin Hospital, Shanghai Jiao Tong University School of Medicine.

### Islets isolation and treatment

Islets of Langerhans were isolated from 8-week-old male Sprague-Dawley rats, C57BL/6 mice or *db/db* mice by using collagenase digestion and separated by density gradient centrifugation. Freshly isolated rat islets were cultured in RPMI 1640 medium containing 5.6 mM glucose and 5% fetal bovine serum (FBS) at 37 °C and 5% CO_2_ for 2–3 h. For RNA-Seq and ChIP-Seq sample preparation, incubated islets were transferred into 6-well plates and cultured in RPMI 1640 medium containing 5.6 mM glucose and 0.25% bovine serum albumin (BSA) in the presence or absence of 3 μM A-485 (MedChemExpress, New Jersey, USA) for 16 h or 6 h.

### RNA sequencing

1 μg total RNA from each sample was used to prepare the sequencing library by KAPA Stranded RNA-Seq Library Prep Kit (Illumina, California, USA). Sequencing was performed on IlluminaHiSeq 4000 for 150 cycles. After quality control, raw sequencing data was pretreated into trimmed data and further compared with *Rattus norvegicus* genome by using Hisat2 software. The differentially expressed genes and transcripts (measured by fragments per kilobase of exon per million reads mapped (FPKM) value) were identified by setting a threshold at fold change ≥2.0, *p*-value <0.001.

### Real-time quantitative PCR

Total RNA was extracted from isolated islets by a total RNA extraction kit (Shanghai Promega, Shanghai, China) and reverse transcribed into cDNA using a reverse transcription kit (Toyobo, Osaka, Japan). RT-qPCR was performed with Applied Biosystems 7300 Real-Time PCR machine (Applied Biosystems, California, USA) using a TB Green Premix Ex Taq (Takara, Shiga, Japan) to quantify the expression of genes of interest. Primer sequences are described in Supplementary Table 3. The expression abundance of genes was normalized to 18 S or beta-actin level in each sample.

### Immunostaining

Mice pancreases were fixed in paraformaldehyde and embedded in paraffin for section at 5 μm thickness. After deparaffinage and rehydration, pancreatic sections were incubated with primary antibodies overnight at 4 °C and detected by fluorescein-labeled secondary antibodies. Antibody information was listed in Supplementary Table 4. Digital images were taken by LSM 880 confocal microscope (Zeiss, Oberkochen, Germany).

### Blood glucose and serum insulin measurement

Blood glucose concentrations were measured by glucometer (Johnson & Johnson, New Jersey, USA) from mice tail vein. For insulin release test, mice were fasted overnight, and glucose was delivered via intraperitoneal injection at a dose of 2 g/kg body weight. Blood samples were taken from retroorbital venous plexus at the indicated time points for serum insulin measurement.

### Insulin secretion assay

Isolated islets were pre-incubated in Krebs-Ringer Buffer (KRB) that contained 3.3 mM glucose and 0.25% BSA at 37 °C for 30 min after indicated treatment. Then, ten islets per assay in triplicate were incubated with prewarmed KRB that contained other additions as indicated for a further 60 min at 37 °C. The supernatant was removed for insulin secretion analysis, and the islets were extracted with acid-ethanol for determination of insulin content. Insulin levels of all samples were estimated by rat/mouse ultrasensitive insulin ELISA kits (ALPCO, Massachusetts, USA).

### Cell culture and treatment

INS-1 cells were cultured in RPMI 1640 medium with 11.1 mM glucose and 10% FBS at 37 °C and 5% CO_2_. For total DNA extraction (Qiagen, Dusseldorf, Germany) and protein extraction, cells were transplanted in 6-well plate and cultured in RPMI 1640 medium containing 5.6 mM glucose and 0.25% BSA in the presence of 3 μM A-485, 10 μg/ml cycloheximide (CHX, Sigma, Missouri, USA), or 10 μM MG132 (Beyotime Biotechnology, Shanghai, China).

### Western blot

Isolated islets or INS-1 cells were treated with RIPA lysis buffer for total protein extraction. Degenerated protein lysates were added into SDS-PAGE gel for electrophoresis and transferred to PVDF membranes. Antibody information was listed in Supplemental Table 4. Horseradish peroxidase (HRP)-conjugated secondary antibodies bound to the primary antibodies were detected by ECL substrate reagents. Digital images were taken by a LAS-4000 Super CCD Remote Control Science Imaging System (Fujifilm, Tokyo, Japan).

### ChIP sequencing

Incubated rat islets were crosslinked with formaldehyde and sonicated to shear the chromatin into appropriate fragments. Immunoprecipitation was performed with H3K27Ac antibody (Millipore, Massachusetts, USA). Sequencing library was prepared using TruSeq Nano DNA Sample Prep Kit (Illumina). Sequencing was performed on IlluminaHiSeq 4000 using HiSeq 3000/4000 SBS Kit for 300 cycles. MACS v1.4.2 (Model-based Analysis of ChIP-seq) software ran with the mapped reads to detect the statistically significant ChIP-enriched peaks compared with respective Input group by a *p-*value threshold of 10^−4^. The differentially enriched peaks between control and A-485 groups were identified by a fold change ≥2.0, *p*-value <0.001. All regions were annotated by the gene whose TSS was nearest to the center of peak region according to the newest UCSC RefSeq database and divided into five classes based on the distance to UCSC RefSeq genes. Data visualization was performed by Integrative Genomics Viewer (IGV).

### Immunoprecipitation

INS-1 cells were treated with 3 μM A-485 for 6 h and harvested for endogenous immunoprecipitation. Protein lysates were incubated with anti-Hnf1α (Proteintech, Illinois, USA) at 4 °C for 2 h and then with Protein A/G PLUS-Agarose beads (Santa Cruz, Texas, USA) overnight at 4 °C. The binding complexes were harvested by centrifugation, washed with lysis buffer, and then eluted with loading buffer. Western blot was followed using anti-p300 (Santa Cruz) and anti-acetyllysine (Cell Signaling, Massachusetts, USA) antibodies for acetylation and binding detection.

### Statistics

Data are presented as Means ± SD. Comparisons were performed by Student’s *t*-test for two groups or ANOVA for multiple groups. Significance was established at *p* < 0.05.

## Supplementary information

Supplementary Information

Figure S1

Figure S2

Figure S3

Table S1

Table S2

Table S3

Table S4

## References

[1] Talchai C, Xuan S, Lin HV, Sussel L, Accili D (2012). Pancreatic β cell dedifferentiation as a mechanism of diabetic β cell failure. Cell.

[2] Swisa A, Glaser B, Dor Y (2017). Metabolic stress and compromised identity of pancreatic beta cells. Front. Genet..

[3] Gu C (2010). Pancreatic beta cells require NeuroD to achieve and maintain functional maturity. Cell Metab..

[4] Taylor BL, Liu FF, Sander M (2013). Nkx6.1 is essential for maintaining the functional state of pancreatic beta cells. Cell Rep..

[5] Gao T (2014). Pdx1 maintains β cell identity and function by repressing an α cell program. Cell Metab..

[6] Nishimura W, Takahashi S, Yasuda K (2015). MafA is critical for maintenance of the mature beta cell phenotype in mice. Diabetologia.

[7] Swisa A (2017). PAX6 maintains β cell identity by repressing genes of alternative islet cell types. J. Clin. Invest.

[8] Jonas JC (1999). Chronic hyperglycemia triggers loss of pancreatic beta cell differentiation in an animal model of diabetes. J. Biol. Chem..

[9] Guo S (2013). Inactivation of specific β cell transcription factors in type 2 diabetes. J. Clin. Invest..

[10] Nordmann TM (2017). The Role of Inflammation in β-cell Dedifferentiation. Sci. Rep..

[11] Efrat S (2019). Concise Review: Beta-cell dedifferentiation in type 2 diabetes. Stem Cells.

[12] Tessarz P, Kouzarides T (2014). Histone core modifications regulating nucleosome structure and dynamics. Nat. Rev. Mol. Cell Biol..

[13] Creyghton MP (2010). Histone H3K27ac separates active from poised enhancers and predicts developmental state. Proc. Natl Acad. Sci. USA.

[14] Heinz S, Romanoski CE, Benner C, Glass CK (2015). The selection and function of cell type-specific enhancers. Nat. Rev. Mol. Cell Biol..

[15] Jin Q (2011). Distinct roles of GCN5/PCAF-mediated H3K9ac and CBP/p300-mediated H3K18/27ac in nuclear receptor transactivation. EMBO J..

[16] Dancy BM, Cole PA (2015). Protein lysine acetylation by p300/CBP. Chem. Rev..

[17] Bedford DC, Kasper LH, Fukuyama T, Brindle PK (2010). Target gene context influences the transcriptional requirement for the KAT3 family of CBP and p300 histone acetyltransferases. Epigenetics.

[18] Sharma A (1999). The NeuroD1/BETA2 sequences essential for insulin gene transcription colocalize with those necessary for neurogenesis and p300/CREB binding protein binding. Mol. Cell Biol..

[19] Qiu Y, Guo M, Huang S, Stein R (2002). Insulin gene transcription is mediated by interactions between the p300 coactivator and PDX-1, BETA2, and E47. Mol. Cell Biol..

[20] Stanojevic V, Habener JF, Thomas MK (2004). Pancreas duodenum homeobox-1 transcriptional activation requires interactions with p300. Endocrinology.

[21] Bonnefond A (2011). Disruption of a novel Kruppel-like transcription factor p300-regulated pathway for insulin biosynthesis revealed by studies of the c.-331 INS mutation found in neonatal diabetes mellitus. J. Biol. Chem..

[22] Ban N (2002). Hepatocyte nuclear factor-1alpha recruits the transcriptional co-activator p300 on the GLUT2 gene promoter. Diabetes.

[23] Fernandez-Zapico ME (2009). MODY7 gene, KLF11, is a novel p300-dependent regulator of Pdx-1 (MODY4) transcription in pancreatic islet beta cells. J. Biol. Chem..

[24] Hussain MA (2006). Increased pancreatic beta-cell proliferation mediated by CREB binding protein gene activation. Mol. Cell Biol..

[25] Wong CK (2018). The p300 and CBP transcriptional coactivators are required for β-cell and α-cell proliferation. Diabetes.

[26] Lasko LM (2017). Discovery of a selective catalytic p300/CBP inhibitor that targets lineage-specific tumours. Nature.

[27] Ediger BN (2017). LIM domain-binding 1 maintains the terminally differentiated state of pancreatic β cells. J. Clin. Invest.

[28] Blum B (2012). Functional beta-cell maturation is marked by an increased glucose threshold and by expression of urocortin 3. Nat. Biotechnol..

[29] Qiu WL (2017). Deciphering pancreatic islet β cell and α cell maturation pathways and characteristic features at the single-cell level. Cell Metab..

[30] Kim-Muller JY (2016). Aldehyde dehydrogenase 1a3 defines a subset of failing pancreatic β cells in diabetic mice. Nat. Commun..

[31] Lemaire K, Thorrez L, Schuit F (2016). Disallowed and allowed gene expression: two faces of mature islet beta cells. Annu Rev. Nutr..

[32] Pullen TJ (2010). Identification of genes selectively disallowed in the pancreatic islet. Islets.

[33] Thorrez L (2011). Tissue-specific disallowance of housekeeping genes: the other face of cell differentiation. Genome Res..

[34] Dhawan S, Georgia S, Tschen S-I, Fan G, Bhushan A (2011). Pancreatic β cell identity is maintained by DNA methylation-mediated repression of Arx. Dev. Cell.

[35] Lu TT-H (2018). The polycomb-dependent epigenome controls β cell dysfunction, dedifferentiation, and diabetes. Cell Metab..

[36] Chen H (2009). Polycomb protein Ezh2 regulates pancreatic beta-cell Ink4a/Arf expression and regeneration in diabetes mellitus. Genes Dev..

[37] Rozzo A, Meneghel-Rozzo T, Delakorda SL, Yang SB, Rupnik M (2009). Exocytosis of insulin: in vivo maturation of mouse endocrine pancreas. Ann. N. Y. Acad. Sci..

[38] Galcheva S, Demirbilek H, Al-Khawaga S, Hussain K (2019). The genetic and molecular mechanisms of congenital hyperinsulinism. Front. Endocrinol..

[39] Martens GA (2007). Specificity in beta cell expression of L-3-hydroxyacyl-CoA dehydrogenase, short chain, and potential role in down-regulating insulin release. J. Biol. Chem..

[40] Lantz KA (2004). Foxa2 regulates multiple pathways of insulin secretion. J. Clin. Invest.

[41] Irles E (2015). Enhanced glucose-induced intracellular signaling promotes insulin hypersecretion: pancreatic beta-cell functional adaptations in a model of genetic obesity and prediabetes. Mol. Cell Endocrinol..

[42] Li C (2010). Mechanism of hyperinsulinism in short-chain 3-hydroxyacyl-CoA dehydrogenase deficiency involves activation of glutamate dehydrogenase. J. Biol. Chem..

[43] Henquin JC, Nenquin M (2018). Immaturity of insulin secretion by pancreatic islets isolated from one human neonate. J. Diabetes Investig..

[44] Dohda T (2004). Transcriptional coactivators CBP and p300 cooperatively enhance HNF-1alpha-mediated expression of the albumin gene in hepatocytes. J. Biochem..

[45] Shih DQ (2001). Loss of HNF-1alpha function in mice leads to abnormal expression of genes involved in pancreatic islet development and metabolism. Diabetes.

[46] Kitamura YI (2005). FoxO1 protects against pancreatic beta cell failure through NeuroD and MafA induction. Cell Metab..

[47] Zhou F (2020). Selective inhibition of CBP/p300 HAT by A-485 results in suppression of lipogenesis and hepatic gluconeogenesis. Cell Death Dis..

[48] Cinti F (2016). Evidence of β-cell dedifferentiation in human type 2 diabetes. J. Clin. Endocrinol. Metab..

[49] Papizan JB (2011). Nkx2.2 repressor complex regulates islet β-cell specification and prevents β-to-α-cell reprogramming. Genes Dev..

[50] Spijker HS (2015). Loss of β-cell identity occurs in type 2 diabetes and is associated with islet amyloid deposits. Diabetes.

[51] Sun J (2019). β-cell dedifferentiation in patients with T2D with adequate glucose control and nondiabetic chronic pancreatitis. J. Clin. Endocrinol. Metab..

[52] Pirot P (2007). Global profiling of genes modified by endoplasmic reticulum stress in pancreatic beta cells reveals the early degradation of insulin mRNAs. Diabetologia.

[53] Hussain MA, Habener JF (1999). Glucagon gene transcription activation mediated by synergistic interactions of pax-6 and cdx-2 with the p300 co-activator. J. Biol. Chem..

[54] Dhawan S (2015). DNA methylation directs functional maturation of pancreatic β cells. J. Clin. Invest..

[55] Narita T, Weinert BT, Choudhary C (2018). Functions and mechanisms of non-histone protein acetylation. Nat. Rev. Mol. Cell Biol..

[56] Eeckhoute J, Formstecher P, Laine B (2001). Maturity-onset diabetes of the young Type 1 (MODY1)-associated mutations R154X and E276Q in hepatocyte nuclear factor 4alpha (HNF4alpha) gene impair recruitment of p300, a key transcriptional co-activator. Mol. Endocrinol..

[57] Malecki MT (1999). Mutations in NEUROD1 are associated with the development of type 2 diabetes mellitus. Nat. Genet..

[58] Matschinsky FM (1996). Banting Lecture 1995. A lesson in metabolic regulation inspired by the glucokinase glucose sensor paradigm. Diabetes.

[59] Camtosun E (2015). A deep intronic HADH splicing mutation (c.636+471G>T) in a congenital hyperinsulinemic hypoglycemia case: long term clinical course. J. Clin. Res Pediatr. Endocrinol..

[60] Miguel-Escalada I (2019). Human pancreatic islet three-dimensional chromatin architecture provides insights into the genetics of type 2 diabetes. Nat. Genet..

