# Supplementary material for: CBP/p300 HAT maintains the gene network critical for β cell identity and functional maturity
Source: Cell Death Dis. 2021 May 12;12(5):476. doi: 10.1038/s41419-021-03761-1 (PMC8116341; doi:10.1038/s41419-021-03761-1)
Supplement: Supplementary file 1 — Supplementary Information [file 41419_2021_3761_MOESM1_ESM.docx]

**Supplementary Information**

Supplementary information includes 3 Figures and 4 Tables.

**Figure S1. No derepression of β cell disallowed genes in A-485-treated rat islets.** (A) Heatmap of β cell disallowed genes identified by Pullen et al [32]. (B) Heatmap of β cell disallowed genes identified by Thorrez et al [33].

**Figure S2. mRNA expression levels of the genes involved in glucose metabolism.** (A) Glycolysis pathway and TCA cycle. Downregulated mRNA expression levels shown in red. Blue presents no change or not detected. (B) Heatmap of the genes involved in glycolysis pathway. (C) Heatmap of the genes involved in pentose phosphate pathway. (D) Heatmap of the genes involved in TCA cycle. (E) Heatmap of the genes involved in oxidative phosphorylation.

**Figure S3. Heatmap of the CHI pathogenic genes in control and A-485-treated rat islets.** Heatmap of the genes involved in the pathogenesis of congenital hyperinsulinism in human.

**Table S1. A-485-regulated genes enriched in β cells.**

**Table S2. A-485-regulated genes enriched in α cells.**

**Table S3. Realtime-PCR primer sequences.**

**Table S4. Antibodies used in this article.**
