# Supplementary figures and images for: CBP/p300 HAT maintains the gene network critical for β cell identity and functional maturity
Source: Cell Death Dis. 2021 May 12;12(5):476. doi: 10.1038/s41419-021-03761-1 (PMC8116341; doi:10.1038/s41419-021-03761-1)

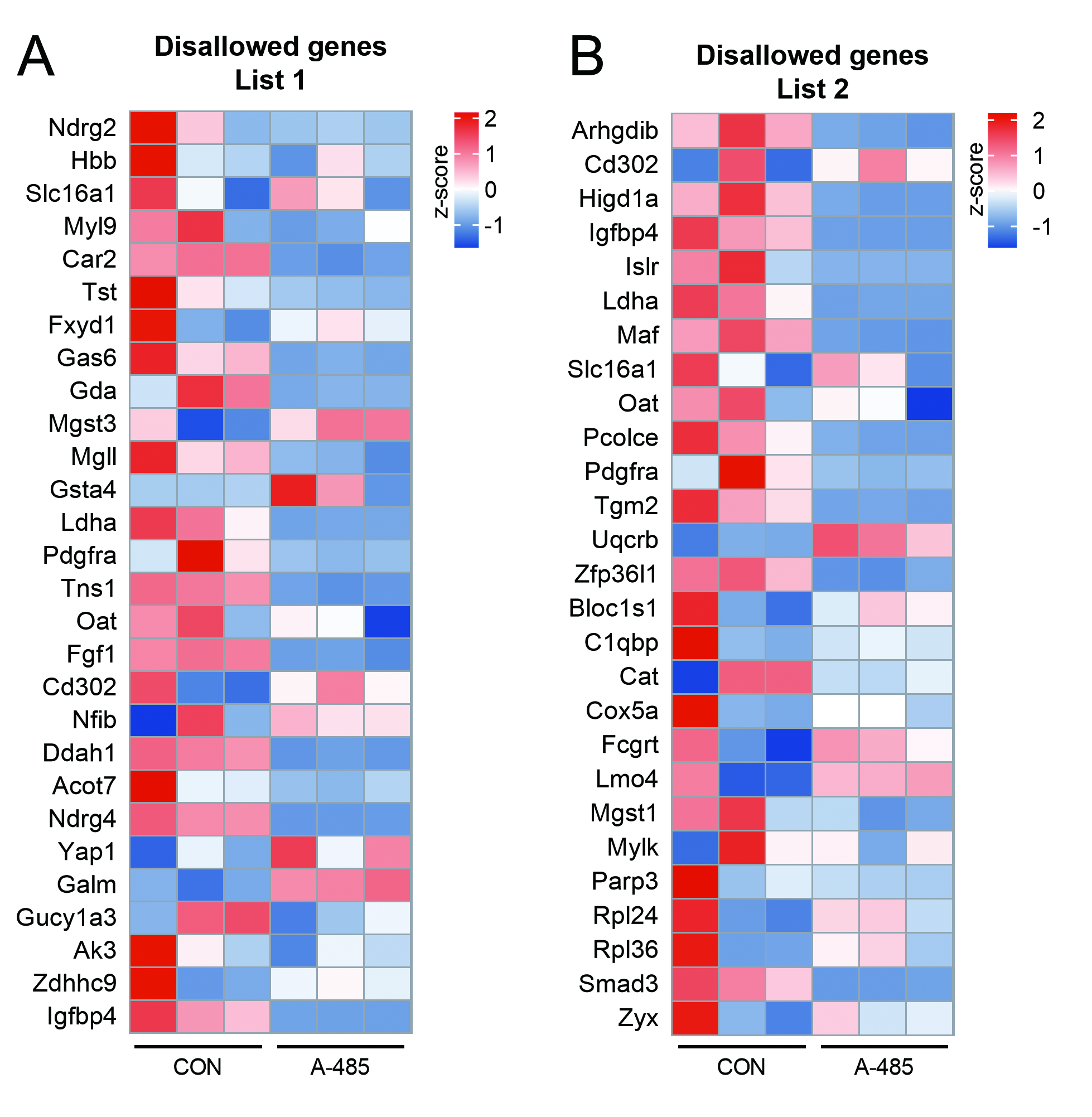

Supplement: Supplementary file 2 — Figure S1 [file 41419_2021_3761_MOESM2_ESM.tif]

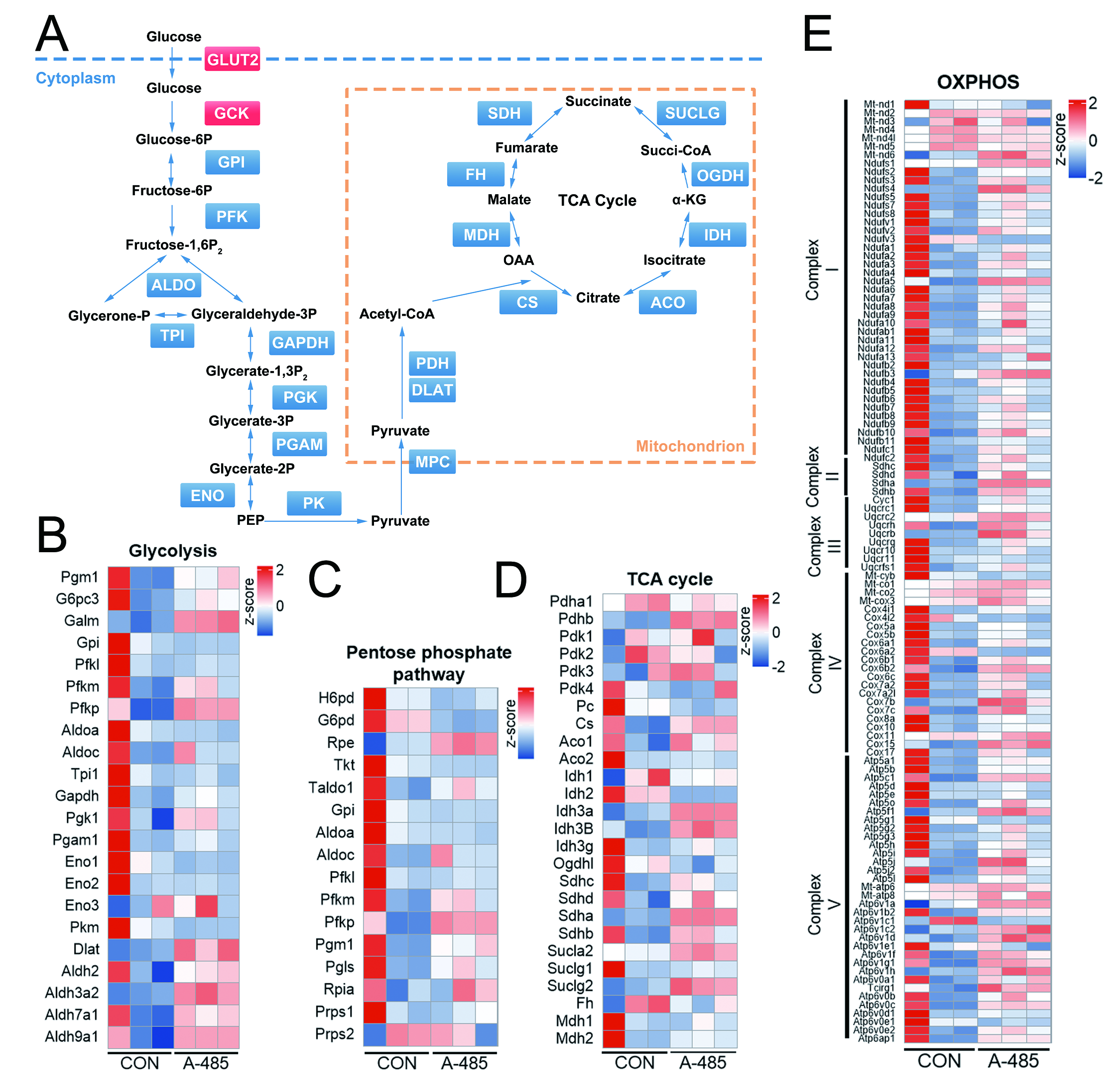

Supplement: Supplementary file 3 — Figure S2 [file 41419_2021_3761_MOESM3_ESM.tif]

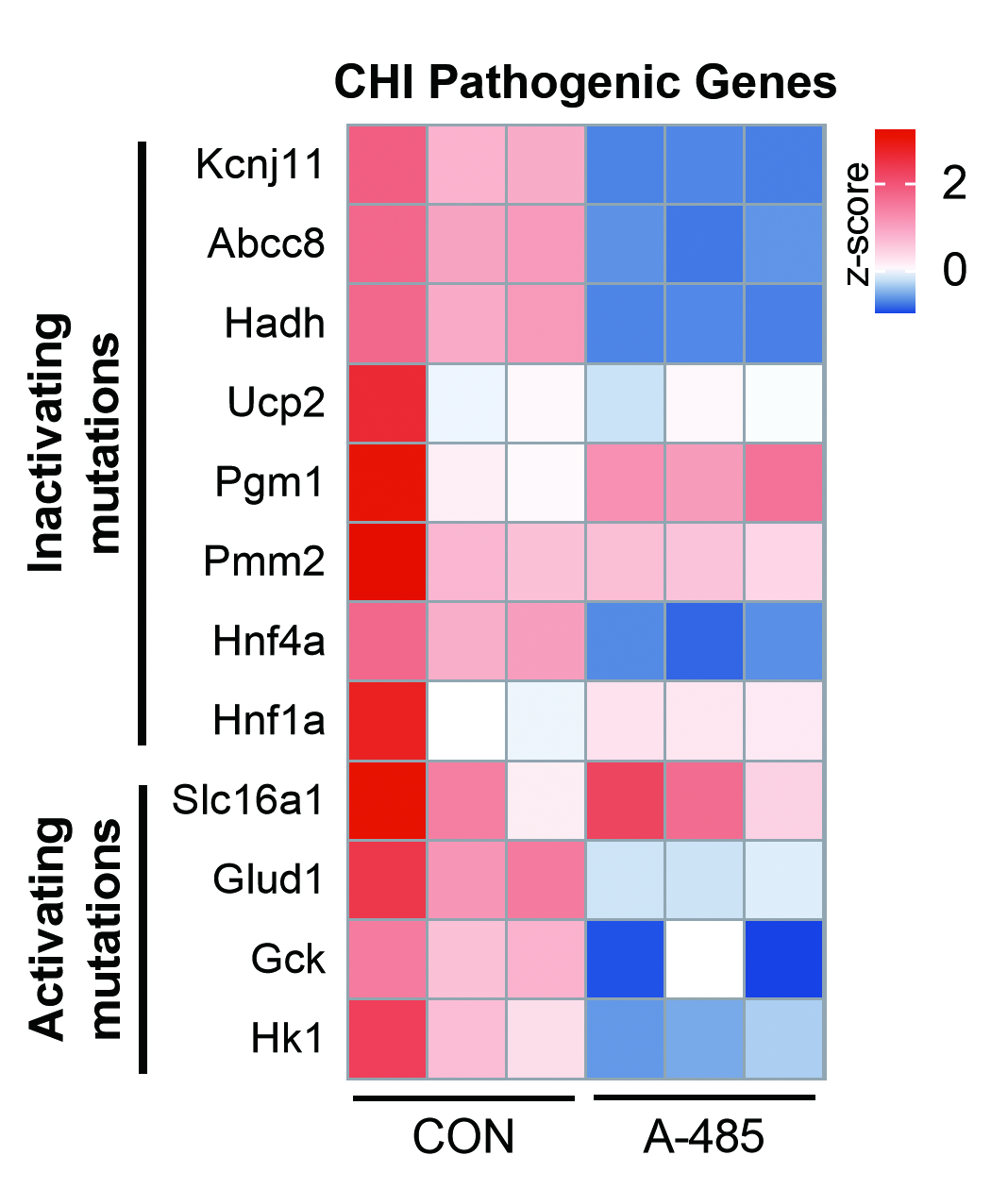

Supplement: Supplementary file 4 — Figure S3 [file 41419_2021_3761_MOESM4_ESM.tif]
